# The Puerto Rico Young Adults’ Stress, Contextual, Behavioral, and Cardiometabolic Risk (PR-OUTLOOK) Study: design and methods

**DOI:** 10.1093/aje/kwae163

**Published:** 2024-06-26

**Authors:** Cynthia M Pérez, Catarina I Kiefe, Sharina D Person, Katherine L Tucker, Polaris Torres, Estefanía Sandoval, Claudia Boneu, Zuleika Ramírez, Josiemer Mattei, José Rodríguez-Orengo, Israel Almodóvar-Rivera, Milagros C Rosal

**Affiliations:** Department of Biostatistics and Epidemiology, Graduate School of Public Health, University of Puerto Rico, Medical Sciences Campus, San Juan, Puerto Rico, United States; Department of Population and Quantitative Health Sciences, University of Massachusetts Chan Medical School, Worcester, MA, United States; Department of Population and Quantitative Health Sciences, University of Massachusetts Chan Medical School, Worcester, MA, United States; Department of Biomedical and Nutritional Sciences, Zuckerberg College of Health Sciences, and Center for Population Health, University of Massachusetts Lowell, Lowell, MA, United States; Department of Biostatistics and Epidemiology, Graduate School of Public Health, University of Puerto Rico, Medical Sciences Campus, San Juan, Puerto Rico, United States; Department of Biostatistics and Epidemiology, Graduate School of Public Health, University of Puerto Rico, Medical Sciences Campus, San Juan, Puerto Rico, United States; Department of Biostatistics and Epidemiology, Graduate School of Public Health, University of Puerto Rico, Medical Sciences Campus, San Juan, Puerto Rico, United States; Department of Biostatistics and Epidemiology, Graduate School of Public Health, University of Puerto Rico, Medical Sciences Campus, San Juan, Puerto Rico, United States; Department of Nutrition, Harvard T.H. Chan School of Public Health, Boston, MA, United States; Fundación de Investigación Clinical Research, San Juan, Puerto Rico, United States; Department of Biochemistry School of Medicine, University of Puerto Rico, Medical Sciences Campus, San Juan, Puerto Rico, United States; Department of Mathematical Sciences, College of Arts and Sciences, University of Puerto Rico Mayaguez Campus, Mayagüez, Puerto Rico, United States; Department of Population and Quantitative Health Sciences, University of Massachusetts Chan Medical School, Worcester, MA, United States

**Keywords:** cardiovascular health, risk factors, prospective cohort study, young adults, Hispanics/Latinos, Puerto Ricans, stress, resiliency

## Abstract

The Puerto Rico (PR) Young Adults’ Stress, Contextual, Behavioral and Cardiometabolic Risk Study (PR-OUTLOOK) is investigating overall and component-specific cardiovascular health (CVH) and cardiovascular disease (CVD) risk factors in a sample of young Puerto Rican adults (aged 18-29 years) in PR (target *n* = 3000) and examining relationships between individual-, family- or social-, and neighborhood-level stress and resilience factors and CVH and CVD risk factors. The study researchers are conducting standardized measurements of CVH and CVD risk factors and demographic, behavioral, psychosocial, neighborhood, and contextual variables and establishing a biorepository of blood, saliva, urine, stool, and hair samples. The assessment methods are aligned with other National Heart, Lung, and Blood Institute–funded studies: the Puerto Rico Observational Study of Psychosocial, Environmental, and Chronic Disease Trends of adults aged 30-75 years; the Hispanic Community Health Study/Study of Latinos; the Boston Puerto Rican Health Study; and the Coronary Artery Risk Development in Young Adults. PR-OUTLOOK data and the study biorepository will facilitate future longitudinal studies of the temporality of associations between stress and resilient factors and CVH and CVD risk factors among young Puerto Ricans. These resources have potential for advancing the scientific understanding of these conditions in a high-risk but understudied young population.

## Introduction

Although cardiovascular disease (CVD) risk has declined among older groups in the US population, CVD risk factors among young adults (typically defined as encompassing the age range of 18-35 years) have increased,^1^ and incident CVD (eg, heart failure) and some CVD outcomes (eg, myocardial infarction) have not declined, or have increased (eg, acute ischemic stroke and CVD mortality).^2^^‑^^5^ Life’s Essential 8, a metric of 4 behavioral (smoking, physical activity, diet quality, and sleep) and 4 health (blood pressure, fasting glucose, non–high-density lipoprotein cholesterol, and body mass index) factors, has been proposed as an overall measure of cardiovascular health (CVH).^6^ Understanding overall CVH, rather than just assessing the presence of individual CVD risk factors, is vital in current young adults—a generation that grew up during the dual epidemic of obesity and type 2 diabetes^7^^,^^8^ and unhealthy lifestyle behaviors.^9^^‑^^20^

Multiple factors may reduce CVH in young adults, including high stress levels (eg, those due to financial, school-to-work, and social/family transitions), a peak in behavioral risks, and undervaluing of health care and illness prevention.^21^^,^^22^ However, CVH and CVD risks have been understudied among young adults, especially Hispanic/Latino adults. The Coronary Artery Risk Development in Young Adults (CARDIA) study, initiated in 1985, examined CVD risk among young Black and White adults but excluded Hispanic/Latino individuals.^23^ The Hispanic Community Health Study/Study of Latinos (HCHS/SOL), a study of adults aged 18-74 years in the continental United States, shows marked variations in risk, prevalence, and outcomes among Hispanic/Latino subgroups, with Puerto Ricans having strikingly greater CVD risk and burden, and poorer overall CVH, than other groups.^10^^,^^24^^‑^^28^ However, little is known regarding CVH and CVD risk factors of young adults residing in Puerto Rico (PR).

Existing literature links psychosocial and contextual stress and CVD risk,^29^^‑^^32^ but these associations have not been studied among young adults in PR. In the past few years, Puerto Ricans have suffered major stressors (ie, a financial crisis, earthquakes, 3 hurricanes, and COVID-19).^33^^‑^^35^ Poverty, present in 39.6% of the population,^36^ may exacerbate these stressors. With young adults facing considerable social, economic, and contextual challenges,^21^ it is critical to understand how these stressors may contribute to CVH. Similarly, it is essential to understand the role of culture-specific factors, such as family and social support, spirituality, and religiosity, as potential protective influences. With atherosclerosis developing well before its clinical manifestation, studies of CVH and CVD risk in young Puerto Ricans may facilitate early CVD prevention.^22^^,^^37^^‑^^47^

This article describes the methods of the PR Young Adults’ Stress, Contextual, Behavioral, and Cardiometabolic Risk (PR-OUTLOOK) study established to address critical gaps in our understanding of CVH and CVD risk factors (Table 1) among young adults in PR. The study objectives are to describe CVH metrics and CVD risk factors in a community sample of young adults in PR and to examine associations between psychosocial and neighborhood stress and CVH metrics and CVD risk factors. Our hypotheses are (1) that stress is negatively associated with CVH metrics and positively associated with CVD risk factors, and (2) resilience factors modify associations of stress and CVH metrics and CVD risk factors.

**Table 1:** Variables assessed at the survey and clinical assessment of the Puerto Rico Young Adults’ Stress, Contextual, Behavioral, and Cardiometabolic Risk Study.

**Domain**	**Variables assessed**
Demographic and socioeconomic characteristics	Age, marital status, children’s ages (if any), and living arrangements Race, nativity, and migration history Skin color: self-described^60^ and objectively measured (Nix Sensor Ltd.) Sex at birth, gender identity, and sexual orientation Education (self and parents), annual household income, work status, health insurance, and health literacy^59^ Municipality of residence Place of residence (urban vs rural) Perceived social standing^55^ Food insecurity^56^ Economic insecurity^57^ Housing instability Material deprivation in childhood^58^
Medical and health care history	Self-reported CVD history (self and family) and other chronic diseases Women’s health questions Perceived health^57^ Medications type, dose, frequency, and reason (prescribed and over the counter) Height and weight (measured); BMI (calculated)^90^ Waist and hip circumferences (measured); waist to hip ratio^90^ Systolic and diastolic blood pressures and heart rate (measured) Total cholesterol, HDL cholesterol, and triglyceride levels (laboratory assays) and estimated LDL cholesterol level Blood glucose, hemoglobin A_1c_, and insulin levels (laboratory assays) High-sensitivity C-reactive protein and fibrinogen levels (laboratory assays) Health care provider access and last provider visit date
Health behaviors	Use of nicotine and substances: Self-reported cigarette smoking and secondhand nicotine exposure^61^ Self-reported vaping frequency, devices, and substances^62^ Self-reported use of nonprescribed medications, stimulants to improve sexual performance, energy drinks, marijuana, and cocaine^62^
	Physical activity: Self-reported physical activity (frequency, duration, and intensity)^63^^,^^64^ Self-reported sedentariness^65^ Objectively measured activity and sedentariness in a subsample (GENEActiv Original device; Activinsights)
	Sleep: Objectively measured (GENEActiv Original device; Activinsights) and self-reported sleep duration and variability (surveys and diary),^66^ quality,^67^^,^^68^ and napping^69^ Self-reported morningness and eveningness,^66^^,^^70^ insomnia,^67^^,^^68^ daytime dysfunction,^71^ and sleep apnea symptoms^66^
	Dietary intake in the past year^92^^,^^93^
	COVID-19–related behaviors
Psychosocial factors	Psychological symptoms: depressive symptoms^27^^,^^72^; anxiety symptoms^73^; post-traumatic stress disorder symptoms^74^; experience of *ataque de nervios*^75^ Stress variables: perceived stress^76^; chronic stressors^77^; traumatic stress from natural disasters^78^; discrimination and unfair treatment^79^; loneliness^80^; caregiving for a sick or frail relative or friend^81^; beliefs about obligations to care for others^57^
	Individual resources: coping^82^; optimism^83^; religiosity^84^; spirituality^85^^,^^86^; social support and strain from partner, family, and friends^87^; life satisfaction^57^; trust in others^57^; pet ownership and type^88^
Neighborhood and contextual factors	Neighborhood conditions^89^: neighborhood violence and safety; neighborhood integration; social cohesion; social interactions with neighbors; aesthetic quality; walking environment; availability of healthy foods
	Contextual factors: Social and political participation^57^ Social relations, support, and reciprocity systems^57^
Outcome measures	Primary outcomes^6^: overall CVH metric score; diet quality metric score; physical activity metric score; nicotine exposure metric score; sleep health metric score; BMI metric score; non–HDL-cholesterol metric score; blood glucose metric score; blood pressure metric score; secondary outcomes (traditional risk factors)^1^: current smoking; physical inactivity; unhealthy diet; overweight or obesity; hypertension; dyslipidemia; diabetes mellitus

Abbreviations: BMI, body mass index; CVD, cardiovascular disease; CVH, cardiovascular health; HDL, high-density lipoprotein; LDL, low-density lipoprotein.

PR-OUTLOOK is guided by a biopsychosocial perspective,^48^ which argues that ethnic health disparities are influenced by differential exposure to psychosocial adversities, with moderation by the level of access to material, emotional, and other resources over time; that an imbalance between stress and resources is created by socioeconomic factors defining the social hierarchy and life opportunities; and that this disadvantaged status is maintained and worsened by debilitating environments of poverty and deprivation.

## Methods

### Study design

PR-OUTLOOK researchers are establishing a cohort of 3000 men and women aged 18-29 years across the island of PR using community-based approaches and conducting comprehensive survey and clinical assessments, followed by brief biannual telephone contacts. A randomly selected sub-sample of 1500 participants, stratified by sex and socioeconomic position index,^49^ completes the actigraphy measures. Recruitment started in September 2020 and will continue for 3.5 years, with biannual contacts projected to end in 2024. The University of Puerto Rico-Medical Sciences Campus Institutional Review Board approved the study.

### Participant eligibility, recruitment, and consent

Eligible individuals are: (1) aged 18 to 29 years; (2) self-identify as Puerto Rican, born in PR, or have at least 1 Puerto Rican–born parent; (3) reside in PR; (4) have phone access; (5) have no cognitive, psychiatric, or physical conditions that preclude participation; (6) are not on active military duty; and (7) have no immediate relatives participating in the study.

Participants are recruited throughout the island via public announcements on traditional and social media; in press releases and electronic and print advertisements; referrals; and community outreach activities. Pre-eligibility is assessed by telephone or online (Research Electronic Data Capture [REDCap] survey),^50^ and recruiters determine final eligibility via telephone. We originally planned a population-based, stratified, random sampling design for recruitment, which the SARS–COVID-19 pandemic made unfeasible. Thus, we reverted to the more opportunistic recruitment strategy just described. Although this change may make our findings potentially less generalizable, our approach is not uncommon in community-based studies^51^^‑^^54^ and will still provide valuable and needed information. Also, we will address potential recruitment bias, as described under “Statistical Analysis.”

Upon confirming eligibility, research staff members conduct consenting procedures. They explain study activities and risks and benefits to participants and obtain written consent (online with secure access, in person, or by mail) and provide ample time for individuals to read the consent form and ask questions.

### Assessment

The study assessment includes a survey and a clinical visit. Variables assessed are listed in Table 1 and procedures are described in the following sub-sections.

#### Survey

The survey assesses demographics, medical and health care history, health behaviors, psychosocial variables, and contextual factors. Participants who complete the survey online (on a computer, tablet, or smartphone) receive an email with a unique access link. Participants who prefer or need (ie, they have no internet access) spoken administration of the survey are offered interviewer-conducted telephone administration.

#### Demographic and socioeconomic characteristics

The survey assesses age, sex at birth, race, nativity, migration history, gender identity, sexual orientation, marital status, children’s ages (if any), living arrangements, educational attainment, work status, annual household income, perceived social standing (according to the MacArthur Scale of Subjective Social Status),^55^ food insecurity (by the 6-item US Department of Agriculture Household Food Security Scale),^56^ current economic^57^ and housing instability, childhood material deprivation,^58^ health insurance and health literacy,^59^ and self-reported skin color.^60^

#### Medical and health care history

Participants complete a checklist of cardiovascular and other chronic diseases they or their first-degree family members have experienced. Those who respond affirmatively to “female” in the question about sex at birth are asked about contraceptive use, history of polycystic ovarian syndrome, and hysterectomy. Other questions include perceived health,^57^ health care provider access, last provider visit date, COVID-19 perceptions, and preventive behaviors.

#### Health behaviors

The survey assesses cigarette smoking and secondhand nicotine exposure,^61^ vaping,^62^ and use of energy drinks, street drugs, and sexual stimulants.^62^ It also includes measures of physical activity (frequency, duration, and intensity),^63^^,^^64^ sedentariness,^65^ and sleep (duration and variability,^66^ quality,^67^^,^^68^ napping,^69^ morningness and eveningness,^66^^,^^70^ sleep problems,^67^ sleep apnea symptoms^66^ and daytime dysfunction).^71^

#### Psychosocial factors

The survey assesses symptoms of depression (via the 10-item Center for Epidemiological Studies of Depression Scale^27^^,^^72^), trait anxiety (10-item Spielberger’s State–Trait Anxiety Inventory^73^), post-traumatic stress (via the 2-item PTSD Checklist for Civilians^74^), and the experience of a cultural syndrome known as *ataque de nervios* (2-item measure from the National Latino and Asian American Survey).^75^

Also assessed are individual-level stressors and resources, including perceived stress (according to the Perceived Stress Scale-4^76^), the experience of individual chronic stressors (via the Chronic Burden Scale^77^), traumatic stress from natural disasters (2018 World Values Survey for PR^78^), perceived discrimination (according to the Everyday Discrimination Scale^79^), loneliness (using the University of California, Los Angeles, Loneliness Scale^80^), caregiving,^81^ and beliefs about caregiving obligations (according to the Norms Toward Obligation and Solidarity Scale^57^). Assessed individual-level resources include stress coping (via the Shift-and-Persist Scale^82^), optimism (using the Revised Life Orientation Test^83^), religiosity (National Institute of Mental Health Collaborative Psychiatric Epidemiology Survey^84^), and spirituality (according to the Daily Spiritual Experience Scale^85^^,^^86^). The survey also assesses social support and strain from spouse/partner, family, and friends (Midlife Development in the United States Survey^87^), overall satisfaction with life, trust in others (using the Social Networks and Social Resources module of the 2017 International Social Survey Programme)^57^), and pet ownership and type.^88^

#### Neighborhood characteristics and resources

The survey assesses 7 social and physical neighborhood environment features that may be associated with CVD risk: violence, safety, aesthetic quality, walking environment, availability of healthy foods, social cohesion, and neighborhood integration (using the Mujahid Neighborhood Health Questionnaire^89^). It also assesses social and political participation (ie, professional, sports, or social groups), social relations, support systems, and reciprocity within these groups (using the Social Networks and Social Resources module of the 2017 International Social Survey Programme^57^).

#### Clinic visit

After completing the survey, participants attend an in-person clinic visit. A mailing provides (1) directions to the clinic; (2) instructions to refrain from eating, smoking or using various substances, and vigorous physical activity on the morning of the visit, to wear light clothing, and to bring all prescribed and over-the-counter medications; and (3) a kit with instructions for a stool sample collection within 24 hours of the participant’s visit. A subsample of participants receives a wrist-worn actigraph (GENEActiv Original) with wear instructions (ie, on nondominant wrist for 7 consecutive days) and a sleep diary to complete.

On the morning of the visit, participants are screened for COVID-19 symptoms (online or by telephone). The clinic visit is conducted by rigorously trained staff and structured as follows: Upon arrival, the research staff welcomes the participant and briefly explains the procedures. The participant’s stool sample is collected and stored at −20°C. Participants who do not bring this sample may provide it at the visit. For the subsample of participants in the sleep study, the research staff collects the actigraph and sleep diary. Participants are instructed to collect a urine specimen in a labeled cup. The voiding time is recorded. Participants provide whole saliva samples in 2 labeled 2 mL cryovials, using the SalivaBio’s passive drool method, and the Saliva Collection Aid (Salimetrics). Height, weight, and waist and hip circumferences are measured using standardized protocols.^90^ Height is measured using a SECA 213 portable stadiometer and rounded to the nearest centimeter. Weight is measured with a Tanita WB800-S Plus Digital Scale and rounded to the nearest tenth kilogram. Using a measuring tape (American Diagnostic Corp.), waist circumference is measured at the level of the umbilicus and hip circumference at the maximal protrusion of the gluteal muscles to the nearest 0.1 cm. All measurements, except weight, are taken twice in a standing position (light clothing, no shoes). After a 10-minute rest period in a sitting position, systolic and diastolic blood pressures are measured by placing an appropriately sized cuff on the participant’s right arm. Three measures are taken at 2-minute intervals using a digital automatic blood pressure monitor (Omron HEM-907XL).

With the participant seated, a certified phlebotomist follows standard protocols and uses precoded (identification no., collection date, and time) tubes to collect 37.7 mL of blood by venipuncture from an arm vein in the following order: 2.7 mL BD Vacutainer (*n* = 1) buffered sodium citrate, 9 mL Vacuette tube (*n* = 1) serum clot activator, 9 mL Vacuette tube (*n* = 1) serum separator clot activator, 4 mL BD Vacutainer (*n* = 3) K2EDTA, 2.5 mL Paxgene blood RNA tube (*n* = 1), and 2.5 mL Paxgene blood DNA tube (*n* = 1). An additional 15.7 mL of blood is collected for quality validation in a random subsample (10% of participants). Strands of hair (*n* = 3-5; 1-3 cm) are cut from the posterior vertex of the participant’s skull,^91^ wrapped in aluminum foil, and placed in an envelope. Additionally, participants are queried about the frequency of hair washing and salon treatments over the previous 3 months, hair care products used, and steroid use.

The NIX Mini 2 color sensor device (Nix Sensor Ltd.) measures skin color. With the participant sitting with arms flat on a table and head roughly in line with the torso, 4 skin color measures are taken by placing the device flat against the skin on the outer arm, inner arm, cheek, and forehead. The device is connected to a smartphone that transfers spectroscopy data directly into the study’s database.

Dietary intake in the previous year is assessed using a food frequency questionnaire adapted and validated for the Puerto Rican population.^92^^,^^93^ This tool is administered using visual aids to facilitate portion size estimation. Analysis of food and nutrients is performed using the Nutrition Data System for Research (University of Minnesota). The research staff also records medication names and doses from the participant’s drug containers or provided pictures of drug containers, and records self-reported reasons for use.

At the end of the visit, research staff verify the completion of all assessments and provide participants a copy of anthropometric, heart rate, and blood pressure measurements. Participants receive a monetary incentive (US$100). They also receive their laboratory test results, a brochure explaining the tests performed, and a thank-you card within 2 weeks.

### Post–clinic visit procedures

Specimens are processed and prepared for transfer. A total of 37.7 mL of blood, and an additional 15.7 mL for a quality assurance sample, are processed for same-day cardiometabolic assays (ie, levels of total cholesterol, high-density lipoprotein cholesterol, triglycerides, fasting glucose, insulin, glycosylated hemoglobin, fibrinogen, and high-sensitivity C- reactive protein) and biorepository specimens. Plasma and serum are separated in a centrifuge within 1 hour of venipuncture, and samples are refrigerated at 8°C or frozen at −20°C until collected by a local clinical reference laboratory. All other specimens, including whole blood, plasma, serum, buffy coat, Paxgene DNA, Paxgene RNA, saliva, urine, stool, and hair, are prepared and transferred to the study’s biorepository at FDI Clinical Research for long-term storage (specimens are stored at −80°C; hair samples are stored at −20°C) (Table 2). For the sleep study subsample, data are extracted from the actigraph, and sleep diaries are scanned and uploaded to REDCap.

**Table 2:** Specimens in the Puerto Rico Young Adults’ Stress, Contextual, Behavioral, and Cardiometabolic Risk Study biorepository.^a^

**Specimen**	**Amount**
Fasting blood	Whole blood: 0.5 mL cryovials (*n* = 6) Plasma: 0.5 mL cryovials (*n* = 6) Serum: 0.5 mL cryovials (*n* = 6) Buffy coat: 1 mL cryovial PaxGene RNA: 2.5 mL Paxgene DNA: 2.5 mL
Saliva	2-mL cryovials (*n* = 2)
Spot urine	1.5-mL cryovials (*n* = 6)
Stool	1.2 tablespoons (18 mL) stored in 20 mL containers
Hair	1-3 cm

^a^ All specimens are stored at −80°C.

### Biannual contacts

At brief biannual calls (5-10 minutes), study staff update participants’ contact information and document any health information, including a new diagnosis of CVD risk factors or events, emergency room visits, CVD-related hospitalizations, or procedures. Any new diagnosis or hospitalization triggers a request for medical records or death certificates for potentially CVD-related events and adjudication.^94^ Because calls are primarily carried out for cohort retention purposes, we do not expect to observe important health changes in this young sample. Any data collected regarding CVD-related events will be used solely for descriptive purposes.

### Cohort retention

To boost the potential for the study cohort to remain prospectively, we use various strategies to maintain participant engagement over time.^95^^,^^96^ At enrollment, the research staff collect contact information for the participant and 3 alternate contacts. Participants receive a toll-free number to facilitate contact with the study staff. To enable assessment completion, we contact participants at different times of the day and week and schedule clinic visits at convenient times. Participants receive reminder calls to complete the survey and to confirm the clinic visit.

We also maintain contact with participants by mailing holiday cards, annual newsletters, and token gifts with the study logo, and by encouraging them to log into the study website, which includes study updates and health information that might interest young adults. To regain contact with participants whose contact information may have changed, we conduct postal tracing and computer searches, mail handwritten letters, call personal contacts provided by the participant, and conduct home visits.

### Training and certification of research staff

Study staff are fully trained in human participant research ethics, rapport building, and interviewing techniques; REDCap; the study procedures described in the manual of operations; and data privacy and security.

### Data quality assurance, management, and requests

Quality control procedures include (1) a manual of operations with study protocols; (2) training and retraining of staff for measurement standardization; (3) periodic checking and recalibrating of equipment and ongoing monitoring of examiner performance; (4) system validation rules for data entry and comprehensive edits after data submission; (5) validation and verification of data collection completion and quality checks; (6) descriptive analyses to identify errors in the data collection process; (7) calculation of invalid rates of food frequency questionnaire records; and (8) review of progress reports to identify and promptly resolve any difficulties. Data are stored in a password-protected database, with access limited to investigators and key data management staff.

In compliance with the National Institute of Health’s 2003 Data Sharing Policy^97^ that existed at the study’s outset, our data-sharing plan includes the creation of deidentified data sets and accompanying documentation, provision for sharing of data used in publications, and release of data to appropriate investigators under the establishment and signing of a data use agreement.

### Statistical analysis

Proportions and means with 95% confidence limits will be used to characterize categorical and continuous measures, respectively, of overall and component-specific CVH metrics (range, 0-100)^6^ and CVD risk factors (presence or absence) at baseline (aim 1). We will compare these measures across categories of socioeconomic indicators and urban and rural habitation status using *z* tests of proportions and means, depending on the nature of the variable. Using generalized linear models, we will examine for aim 2 the associations between psychosocial and neighborhood stress and CVH metrics and CVD risk factors. These models will be adjusted for age, sex at birth, and individual socioeconomic status, and will use the appropriate canonical link depending on the nature of the data. Directed acyclic graphs will be used to inform the choice of other covariates in the models.^98^^,^^99^ We will also assess the impact of the time lag between the survey and clinical assessment on time-dependent variables and use this as a variable in sensitivity analysis and adjustments.

To assess the modifying effect of resilience factors on associations observed in aim 2, we will introduce these factors as interaction terms with stress (psychosocial and neighborhood) to determine the impact on CVH metrics and CVD risk factors. If the interactions are significant, we will conduct subgroup analyses. All analyses will be performed using STATA (Stata Corp.) and R (R Core Team) software.

Because selection bias may influence key characteristics in this nonprobabilistic sampling, we designed a plan to compare the sample demographics against demographics from the 2020 US Census data for PR, to make corrections to our recruitment strategy. Particular attention will be given to age, sex, and socioeconomic indicators. To correct differences between the final sample and Census data, and to improve generalizability, we will use the statistical approach of iterative proportional fitting, also known as raking.^100^^,^^101^ In this approach, marginal totals from our primary covariates of interest (age, sex, and socioeconomic indicators) in the sample are compared with Census data. Iterative proportional fitting iteratively adjusts the weight for each observation until the sample distribution aligns with the Census data for those variables. Using these weights that directly adjust counts in primary covariates of interest in our sample to those in the Census data will facilitate adjustment of results to approximate those of the target population and allow us to obtain prevalence estimates of CVD risk factors.

#### Statistical power

Because our plan was to develop a cohort, we based our power considerations on the preset sample size of 3000. We used a minimum detectable effect approach. For categorical measures, we conservatively assumed the base proportion of 0.5, the middle of the binomial distribution, where variability is highest. Because our original design included complex survey sampling, we also accounted for a design effect (DEFF), which is the ratio of the sampling variance obtained using a complex design relative to the variance that would have been obtained from a simple random sample without replacement.^102^ We calculated an effective sample size by dividing the available sample by the DEFF and used this to determine the minimum detectable effect. The DEFF was allowed to vary based on the expected mean cluster size. Because the new design no longer uses complex survey sampling, the DEFF is no longer needed, and the available and effective sample sizes are the same.

For aim 1, a sample size of 3000 produces a 2-sided 95% CI with a width equal to 0.036 when the sample proportion is 0.5 (a 95% CI as tight as 0.482-0.518). For aim 2, our comparisons of interest include 4 possible scenarios: continuous predictor and outcome; categorical predictor and outcome; categorical predictor and continuous outcome; and continuous predictor and categorical outcome. For simplicity, we only present power consideration for the first scenario. Although we will use more complex approaches to analyze the data, using more straightforward power calculation approaches with fewer assumptions is commonly accepted. For 2 continuous variables (eg, stress and body mass index), we will have 80% power to detect a correlation as small as 0.05 in the main study (*n* = 3000) and 0.09 in the sleep study (*n* = 1000; ie, actigraphy and survey measures), based on a 2-sided linear regression test with *r* = 0 for 1 normally distributed covariate *X*, with *α* = .05.

For aim 3, there are numerous possible interactions. Therefore, we focused on the impact of subgroup analysis on the minimum detectable effect. An example is the possible modification of the relationship between stress and CVH (both continuous) by functional support (operationalized here as 2 equal-sized groups). Using a similar approach as aim 2 but reducing the sample size to 1500 (1 level of functional support), we will have 80% power to detect a correlation as small as 0.07. If functional support is categorized into, for example, 4 groups (*n* = 750 in each group), we will still have 80% power to detect a correlation as small as 0.10.

### Preliminary results

Because recruitment is near completion, initial analyses were conducted to assess potential selection bias by describing the baseline demographics of the first 2744 participants along with the demographic characteristics of a similarly aged sample from the US Census 2021 PR Community Survey (Table 3).^103^ Data show that our sample has more younger adults, women, married individuals, and privately insured adults than in the Census. Any differences in sociodemographic characteristics between the final sample and Census data will be corrected using the statistical approach of iterative proportional fitting.

**Table 3:** Comparison of baseline characteristics of the first 2744 Puerto Rico Young Adults’ Stress, Contextual, Behavioral, and Cardiometabolic Risk Study respondents with the 2021 Puerto Rico community survey 1-year sample estimates for the population aged 18-29 years in Puerto Rico.^111^

**Sociodemographic characteristic**	**PR-OUTLOOK (*n* = 2744)** **No. (%)**	**Puerto Rico (*n* = 516 047)** **No. (%)**
Age group, years 18-24 25-29	1990 (72.5) 754 (27.5)	311 747 (60.4) 204 300 (39.6)
Sex at birth Male Female	1059 (38.6) 1685 (61.4)	259 472 (50.3) 256 575 (49.7)
Educational attainment High school or less More than high school Missing data	991 (36.1) 1751 (63.8) 2 (0.1)	162 838 (31.6) 353 209 (68.4)
Marital status Married/living with a partner Widowed, divorced, or separated Never married	364 (13.3) 15 (0.5) 2365 (86.2)	34 901 (6.8) 6326 (1.2) 474 820 (92.0)
Health care coverage Private Government programs None Missing data	1629 (59.4) 1025 (37.4) 75 (2.7) 15 (0.5)	217 356 (42.1) 260 101 (50.4) 38 590 (7.5)
Municipality of residence San Juan Other	412 (15.0) 2332 (85.0)	53 669 (10.4) 462 378 (89.6)

Abbreviation: PR-OUTLOOK, Puerto Rico Young Adults’ Stress, Contextual, Behavioral and Cardiometabolic Risk Study.

## Discussion

Previous studies of Puerto Ricans have shown that this population experiences a considerable burden of cardiovascular disparities.^24^^,^^104^^‑^^109^ However, these studies have primarily focused on middle-aged and older adults. To our knowledge, PR-OUTLOOK is the largest and only study inclusive of young adults throughout PR. It will comprehensively assess the overall CVH profile and CVD risk factors of a community sample of 3000 young adults. The study will describe behavioral and cardiometabolic risk factors early, during young adulthood, before they develop or progress to clinical CVD. PR-OUTLOOK will also evaluate associations between psychosocial and neighborhood-level factors and CVH and CVD risk factors in this sample.

The results of PR-OUTLOOK will provide targets for early CVD prevention and prioritization of public health programs, help generate research questions for future longitudinal cohort follow-up, and inform future CVD prevention interventions. Our rich data set and comprehensive biorepository will offer opportunities to evaluate countless scientific hypotheses, furthering insights into the complex interactions between psychosocial and environmental determinants, behaviors, and CVH of young adults. Future longitudinal observations of this cohort will help resolve questions about the temporality of associations between individual- and neighborhood stresses, resilient factors, sociodemographic variables, and behavioral and CVH and CVD risk. The study also lays a solid foundation for comparing young, middle-aged, and older adults on the island (comparisons with the Puerto Rico Observational Study of Psychosocial, Environmental, and Chronic Disease Trends [PROSPECT])^54^ and in the United States (comparisons with HCHS/SOL^61^ and Boston Puerto Rican Health Study^104^) facilitated by shared methods and measures. These comparisons will provide insights for understanding both health advantages and disparities of young adults living in the continental US versus PR, with distinct migration and acculturation dynamics and social and environmental conditions. Research capacity in PR will be strengthened by providing specialized training to research staff and opportunities for hands-on research experience for students and early career investigators, predominantly from underrepresented minoritized groups, as stated in Objective 8 of the National Heart, Lung, and Blood Institute’s strategic vision.^110^

## Acknowledgments

We acknowledge the contributions of the Hispanic Alliance for Clinical and Translational Research (National Institute of General Medical Sciences grant U54GM133807), the community partner clinics, the study participants, and the PR-OUTLOOK research staff and assistants.

## Data Availability

PR-OUTLOOK data will be available from the study’s principal investigators (C.M.P. and M.C.R.) upon reasonable request and compliance with the study’s data request processes.

## References

[1] Virani SS, Alonso A, Aparicio HJ, et al. Heart disease and stroke statistics-2021 update: a report from the American Heart Association. *Circulation*. 2021;143(8):e254-e743. 10.1161/CIR.0000000000000950 33501848 PMC13036842

[2] Andersson C, Vasan RS. Epidemiology of cardiovascular disease in young individuals. *Nat Rev Cardiol*. 2018;15(4):230-240. 10.1038/nrcardio.2017.154 29022571

[3] Gupta A, Wang Y, Spertus JA, et al. Trends in acute myocardial infarction in young patients and differences by sex and race, 2001 to 2010. *J Am Coll Cardiol*. 2014;64(4):337-345. 10.1016/j.jacc.2014.04.054 25060366 PMC4415523

[4] Sipila JOT, Posti JP, Ruuskanen JO, et al. Stroke hospitalization trends of the working-aged in Finland. *PLoS One*. 2018;13(8):e0201633. 10.1371/journal.pone.0201633 30067825 PMC6070270

[5] Ritchey MD, Wall HK, George MG, et al. US trends in premature heart disease mortality over the past 50 years: where do we go from here? *Trends Cardiovasc Med*. 2020;30(6):364-374. 10.1016/j.tcm.2019.09.005 31607635 PMC7098848

[6] Lloyd-Jones DM, Allen NB, Anderson CAM, et al. Life's essential 8: updating and enhancing the American Heart Association's construct of cardiovascular health: a presidential advisory from the American Heart Association. *Circulation*. 2022;146(5):e18-e43. 10.1161/CIR.0000000000001078 35766027 PMC10503546

[7] Lawrence E, Hummer RA, Harris KM. The cardiovascular health of young adults: disparities along the urban-rural continuum. *Ann Am Acad Pol Soc Sci*. 2017;672(1):257-281. 10.1177/0002716217711426 28694547 PMC5501485

[8] Hussey JM, Nguyen QC, Whitsel EA, et al. The reliability of in-home measures of height and weight in large cohort studies: evidence from add health. *Demogr Res*. 2015;32:1081-1098. 10.4054/DemRes.2015.32.39 26146486 PMC4487879

[9] Deshmukh-Taskar PR, O'Neil CE, Nicklas TA, et al. Dietary patterns associated with metabolic syndrome, sociodemographic and lifestyle factors in young adults: the Bogalusa Heart Study. *Public Health Nutr*. 2009;12(12):2493-2503. 10.1017/S1368980009991261 19744354 PMC2792890

[10] Perreira KM, Gotman N, Isasi CR, et al. Mental health and exposure to the United States: key correlates from the Hispanic Community Health Study of Latinos. *J Nerv Ment Dis*. 2015;203(9):670-678. 10.1097/NMD.0000000000000350 26237134 PMC4552578

[11] Deshmukh-Taskar P, Nicklas TA, Yang SJ, et al. Does food group consumption vary by differences in socioeconomic, demographic, and lifestyle factors in young adults? The Bogalusa Heart Study. *J Am Diet Assoc*. 2007;107(2):223-234. 10.1016/j.jada.2006.11.004 17258958 PMC2769987

[12] Nicklas TA, Myers L, Reger C, et al. Impact of breakfast consumption on nutritional adequacy of the diets of young adults in Bogalusa, Louisiana: ethnic and gender contrasts. *J Am Diet Assoc*. 1998;98(12):1432-1438. 10.1016/S0002-8223(98)00325-3 9850113

[13] Bell S, Lee C. Emerging adulthood and patterns of physical activity among young Australian women. *Int J Behav Med*. 2005;12(4):227-235. 10.1207/s15327558ijbm1204_3 16262541

[14] McVeigh JA, Howie EK, Zhu K, et al. Organized sport participation from childhood to adolescence is associated with bone mass in young adults from the Raine Study. *J Bone Miner Res*. 2019;34(1):67-74. 10.1002/jbmr.3583 30328145

[15] Zitting KM, Munch MY, Cain SW, et al. Young adults are more vulnerable to chronic sleep deficiency and recurrent circadian disruption than older adults. *Sci Rep*. 2018;8(1):11052. 10.1038/s41598-018-29358-x 30038272 PMC6056541

[16] Adams RJ, Appleton SL, Taylor AW, et al. Sleep health of Australian adults in 2016: results of the 2016 sleep Health Foundation national survey. *Sleep Health*. 2017;3(1):35-42. 10.1016/j.sleh.2016.11.005 28346149

[17] Wills TA, Knight R, Williams RJ, et al. Risk factors for exclusive e-cigarette use and dual e-cigarette use and tobacco use in adolescents. *Pediatrics*. 2015;135(1):e43-e51. 10.1542/peds.2014-0760 25511118 PMC4279062

[18] Glasser A, Abudayyeh H, Cantrell J, et al. Patterns of e-cigarette use among youth and young adults: review of the impact of e-cigarettes on cigarette smoking. *Nicotine Tob Res*. 2019;21(10):1320-1330. 10.1093/ntr/nty103 29788314

[19] George MG, Tong X, Bowman BA. Prevalence of cardiovascular risk factors and strokes in younger adults. *JAMA Neurol*. 2017;74(6):695-703. 10.1001/jamaneurol.2017.0020 28395017 PMC5559660

[20] Bucholz EM, Gooding HC, de Ferranti SD. Awareness of cardiovascular risk factors in U.S. young adults aged 18-39 years. *Am J Prev Med*. 2018;54(4):e67-e77. 10.1016/j.amepre.2018.01.022 29433955 PMC5893222

[21] Institute of Medicine, National Research Council of the National Academies. *Investing in the Health and Well-Being of Young Adults*. The National Academies Press; 2015. 25855847

[22] Gooding HC, Gidding SS, Moran AE, et al. Challenges and opportunities for the prevention and treatment of cardiovascular disease among young adults: report from a National Heart, Lung, and Blood Institute Working Group. *JAMA*. 2020;9(19):e016115. 10.1161/JAHA.120.016115 PMC7792379 32993438

[23] Friedman GD, Cutter GR, Donahue RP, et al. CARDIA: study design, recruitment, and some characteristics of the examined subjects. *J Clin Epidemiol*. 1988;41(11):1105-1116. 10.1016/0895-4356(88)90080-7 3204420

[24] Daviglus ML, Talavera GA, Aviles-Santa ML, et al. Prevalence of major cardiovascular risk factors and cardiovascular diseases among Hispanic/Latino individuals of diverse backgrounds in the United States. *JAMA*. 2012;308(17):1775-1784. 10.1001/jama.2012.14517 23117778 PMC3777250

[25] Isasi CR, Ayala GX, Sotres-Alvarez D, et al. Is acculturation related to obesity in Hispanic/Latino adults? Results from the Hispanic Community Health Study/Study of Latinos. *J Obes*. 2015;2015(186276):1-8. 10.1155/2015/186276 PMC4393894 25893114

[26] Salazar CR, Strizich G, Seeman TE, et al. Nativity differences in allostatic load by age, sex, and Hispanic background from the Hispanic Community Health Study/Study of Latinos. *SSM Popul Health*. 2016;2:416-424. 10.1016/j.ssmph.2016.05.003 27540567 PMC4985030

[27] Gonzalez P, Nunez A, Merz E, et al. Measurement properties of the Center for Epidemiologic Studies Depression Scale (CES-D 10): findings from HCHS/SOL. *Psychol Assess*. 2017;29(4):372-381. 10.1037/pas0000330 27295022 PMC5154787

[28] Rodriguez F, Lee UJ, Barone N, et al. Risk factor control across the spectrum of cardiovascular risk: findings from the Hispanic Community Health Study/Study of Latinos (HCHS/SOL). *Am J Prev Cardiol*. 2021;5:100147. 10.1016/j.ajpc.2021.100147 34327490 PMC8315414

[29] Williams RB . Psychosocial and biobehavioral factors and their interplay in coronary heart disease. *Annu Rev Clin Psychol*. 2008;4(1):349-365. 10.1146/annurev.clinpsy.4.022007.141237 17716037

[30] Rosengren A, Hawken S, Ounpuu S, et al. Association of psychosocial risk factors with risk of acute myocardial infarction in 11119 cases and 13648 controls from 52 countries (the INTERHEART study): case-control study. *Lancet*. 2004;364(9438):953-962. 10.1016/S0140-6736(04)17019-0 15364186

[31] Everson-Rose SA, Lewis TT. Psychosocial factors and cardiovascular diseases. *Annu Rev Public Health*. 2005;26(1):469-500. 10.1146/annurev.publhealth.26.021304.144542 15760298

[32] Everson-Rose SA, Lutsey PL, Roetker NS, et al. Perceived discrimination and incident cardiovascular events: the Multi-Ethnic Study of Atherosclerosis. *Am J Epidemiol*. 2015;182(3):225-234. 10.1093/aje/kwv035 26085044 PMC4517694

[33] Santos-Burgoa C, Sandberg J, Suarez E, et al. Differential and persistent risk of excess mortality from hurricane Maria in Puerto Rico: a time-series analysis. *Lancet Planet Health*. 2018;2(11):e478-e488. 10.1016/S2542-5196(18)30209-2 30318387

[34] Orengo-Aguayo R, Stewart RW, de Arellano MA, et al. Disaster exposure and mental health among Puerto Rican youths after hurricane Maria. *JAMA Netw Open*. 2019;2(4):e192619. 10.1001/jamanetworkopen.2019.2619 31026024 PMC6487632

[35] Garcia C, Rivera FI, Garcia MA, et al. Contextualizing the COVID-19 era in Puerto Rico: compounding disasters and parallel pandemics. *J Gerontol B Psychol Sci Soc Sci*. 2021;76(7):e263-e267. 10.1093/geronb/gbaa186 33112945 PMC7665778

[36] US Census Bureau . Poverty status in the past 12 months: 2023. American Community Survey 1-year estimates. Accessed March 15, 2023. https://data.census.gov/table?q=poverty%20status%20puerto%20rico

[37] McMahan CA, Gidding SS, McGill HC Jr. Coronary heart disease risk factors and atherosclerosis in young people. *J Clin Lipidol*. 2008;2(3):118-126. 10.1016/j.jacl.2008.02.006 21291730

[38] Berenson GS, Srinivasan SR, Bao W, et al. Association between multiple cardiovascular risk factors and atherosclerosis in children and young adults. The Bogalusa Heart Study. *N Engl J Med*. 1998;338(23):1650-1656. 10.1056/NEJM199806043382302 9614255

[39] Relationship of atherosclerosis in young men to serum lipoprotein cholesterol concentrations and smoking. A preliminary report from the Pathobiological Determinants of Atherosclerosis in Youth (PDAY) Research Group. *JAMA*. 1990;264(23):3018-3024. 10.1001/jama.1990.03450230054029 2243430

[40] Newman WP 3rd, Freedman DS, Voors AW, et al. Relation of serum lipoprotein levels and systolic blood pressure to early atherosclerosis. The Bogalusa Heart Study. *N Engl J Med*. 1986;314(3):138-144. 10.1056/NEJM198601163140302 3455748

[41] McGill HC Jr, McMahan CA, Malcom GT, et al. Effects of serum lipoproteins and smoking on atherosclerosis in young men and women. *Arterioscler Thromb Vasc Biol*. 1997;17(1):95-106. 10.1161/01.ATV.17.1.95 9012643

[42] Mahoney LT, Burns TL, Stanford W, et al. Coronary risk factors measured in childhood and young adult life are associated with coronary artery calcification in young adults: the Muscatine Study. *J Am Coll Cardiol*. 1996;27(2):277-284. 10.1016/0735-1097(95)00461-0 8557894

[43] Raitakari OT, Juonala M, Kahonen M, et al. Cardiovascular risk factors in childhood and carotid artery intima-media thickness in adulthood: the Cardiovascular Risk in Young Finns Study. *JAMA*. 2003;290(17):2277-2283. 10.1001/jama.290.17.2277 14600186

[44] Davis PH, Dawson JD, Riley WA, et al. Carotid intimal-medial thickness is related to cardiovascular risk factors measured from childhood through middle age: the Muscatine Study. *Circulation*. 2001;104(23):2815-2819. 10.1161/hc4601.099486 11733400

[45] Tonstad S, Joakimsen O, Stensland-Bugge E, et al. Risk factors related to carotid intima-media thickness and plaque in children with familial hypercholesterolemia and control subjects. *Arterioscler Thromb Vasc Biol*. 1996;16(8):984-991. 10.1161/01.ATV.16.8.984 8696963

[46] Anyfantakis D, Symvoulakis EK, Panagiotakos DB, et al. Impact of religiosity/spirituality on biological and preclinical markers related to cardiovascular disease. Results from the SPILI III Study. *Hormones (Athens)*. 2013;12(3):386-396. 10.1007/BF03401304 24121380

[47] Mishra SK, Togneri E, Tripathi B, et al. Spirituality and religiosity and its role in health and diseases. *J Relig Health*. 2017;56(4):1282-1301. 10.1007/s10943-015-0100-z 26345679

[48] Myers HF . Ethnicity- and socio-economic status-related stresses in context: an integrative review and conceptual model. *J Behav Med*. 2009;32(1):9-19. 10.1007/s10865-008-9181-4 18989769

[49] Torres-Cintron M, Ortiz AP, Ortiz-Ortiz KJ, et al. Using a socioeconomic position index to assess disparities in cancer incidence and mortality, Puerto Rico, 1995-2004. *Prev Chronic Dis*. 2012;9:E15. 10.5888/pcd9.100271 22172182 PMC3298767

[50] Harris PA, Taylor R, Thielke R, et al. Research electronic data capture (REDCap)--a metadata-driven methodology and workflow process for providing translational research informatics support. *J Biomed Inform*. 2009;42(2):377-381. 10.1016/j.jbi.2008.08.010 18929686 PMC2700030

[51] National Institutes of Health . All of Us Research Program. Accessed August 3, 2023. https://allofus.nih.gov/.

[52] The Women's Health Initiative Study Group . Design of the Women's Health Initiative clinical trial and observational study. *Control Clin Trials*. 1998;19(1):61-109. 10.1016/S0197-2456(97)00078-0 9492970

[53] Sowers M, Crawford S, Sternfeld B, et al. SWAN: a multi-center, multi-ethnic, community-based cohort study of women and the menopausal transition. In: Lobo RA, Kelsey J, Marcus R, eds. *Menopause: Biology and Pathobiology*. Academic Press; 2000:175-188.

[54] Mattei J, Tucker KL, Falcon LM, et al. Design and implementation of the Puerto Rico observational study of psychosocial, environmental, and chronic disease trends (PROSPECT). *Am J Epidemiol*. 2021;190(5):707-717. 10.1093/aje/kwaa231 33083832 PMC8096477

[55] Adler NE, Epel ES, Castellazzo G, et al. Relationship of subjective and objective social status with psychological and physiological functioning: preliminary data in healthy white women. *Health Psychol*. 2000;19(6):586-592. 10.1037/0278-6133.19.6.586 11129362

[56] Blumberg SJ, Bialostosky K, Hamilton WL, et al. The effectiveness of a short form of the household food security scale. *Am J Public Health*. 1999;89(8):1231-1234. 10.2105/AJPH.89.8.1231 10432912 PMC1508674

[57] Joye D, Sapin M, Wolf C. *Measuring Social Networks and Social Resources: An Exploratory ISSP Survey Around the World*. Vol. 22. GESIS - Leibniz-Institut für Sozialwissenschaften; 2019.

[58] Isasi CR, Jung M, Parrinello CM, et al. Association of childhood economic hardship with adult height and adult adiposity among Hispanics/Latinos. The HCHS/SOL Socio-Cultural Ancillary Study. *PLoS One*. 2016;11(2):e0149923. 10.1371/journal.pone.0149923 26919283 PMC4769180

[59] Sarkar U, Schillinger D, Lopez A, et al. Validation of self-reported health literacy questions among diverse English and Spanish-speaking populations. *J Gen Intern Med*. 2011;26(3):265-271. 10.1007/s11606-010-1552-1 21057882 PMC3043178

[60] Palloni A, Luisa Davila A, Sanchez-Ayendez M. Puerto Rican Elderly: Health Conditions (PREHCO) Project, 2002-2003, 2006-2007. Inter-university Consortium for Political and Social Research; 2013.

[61] Sorlie PD, Aviles-Santa LM, Wassertheil-Smoller S, et al. Design and implementation of the Hispanic Community Health Study/Study of Latinos. *Ann Epidemiol*. 2010;20(8):629-641. 10.1016/j.annepidem.2010.03.015 20609343 PMC2904957

[62] Moscoso Álvarez MR, Colón Jordán HM, Reyes Pulliza JC, et al. El Uso De Substancias En Los Escolares Puertorriqueños: Análisis Por Áreas De Servicio De Prevención, Consulta Juvenil 2018-2020. Administración de Servicios de Salud Mental y Contra la Adicción; 2020.

[63] Johnson-Kozlow M, Rock CL, Gilpin EA, et al. Validation of the WHI brief physical activity questionnaire among women diagnosed with breast cancer. *Am J Health Behav*. 2007;31(2):193-202. 10.5993/AJHB.31.2.8 17269909

[64] Meyer AM, Evenson KR, Morimoto L, et al. Test-retest reliability of the Women's Health Initiative physical activity questionnaire. *Med Sci Sports Exerc*. 2009;41(3):530-538. 10.1249/MSS.0b013e31818ace55 19204598 PMC2692735

[65] Rosenberg DE, Norman GJ, Wagner N, et al. Reliability and validity of the sedentary behavior questionnaire (SBQ) for adults. *J Phys Act Health*. 2010;7(6):697-705. 10.1123/jpah.7.6.697 21088299

[66] Quan SF, Howard BV, Iber C, et al. The Sleep Heart Health Study: design, rationale, and methods. *Sleep*. 1997;20(12):1077-1085. 9493915

[67] Levine DW, Kaplan RM, Kripke DF, et al. Factor structure and measurement invariance of the Women's Health Initiative insomnia rating scale. *Psychol Assess*. 2003;15(2):123-136. 10.1037/1040-3590.15.2.123 12847773

[68] Levine DW, Kripke DF, Kaplan RM, et al. Reliability and validity of the Women's Health Initiative insomnia rating scale. *Psychol Assess*. 2003;15(2):137-148. 10.1037/1040-3590.15.2.137 12847774

[69] Naska A, Oikonomou E, Trichopoulou A, et al. Siesta in healthy adults and coronary mortality in the general population. *Arch Intern Med*. 2007;167(3):296-301. 10.1001/archinte.167.3.296 17296887

[70] Horne JA, Ostberg O. A self-assessment questionnaire to determine morningness-eveningness in human circadian rhythms. *Int J Chronobiol*. 1976;4(2):97-110. 1027738

[71] Buysse DJ, Yu L, Moul DE, et al. Development and validation of patient-reported outcome measures for sleep disturbance and sleep-related impairments. *Sleep*. 2010;33(6):781-792. 10.1093/sleep/33.6.781 20550019 PMC2880437

[72] Andresen EM, Malmgren JA, Carter WB, et al. Screening for depression in well older adults: evaluation of a short form of the CES-D (Center for Epidemiologic Studies Depression Scale). *Am J Prev Med*. 1994;10(2):77-84. 10.1016/S0749-3797(18)30622-6 8037935

[73] Spielberger CD, Gorsuch RL, Lushene R, et al. Manual for the State-Trait Anxiety Inventory. Consulting Psychologists Press; 1983.

[74] Lang AJ, Stein MB. An abbreviated PTSD checklist for use as a screening instrument in primary care. *Behav Res Ther*. 2005;43(5):585-594. 10.1016/j.brat.2004.04.005 15865914

[75] Guarnaccia PJ, Lewis-Fernandez R, Martinez Pincay I, et al. Ataque de nervios as a marker of social and psychiatric vulnerability: results from the NLAAS. Int J Soc Psychiatry. 2010;56(3):298-309. 10.1177/0020764008101636 19592438 PMC2865565

[76] Cohen S, Kamarck T, Mermelstein R. A global measure of perceived stress. *J Health Soc Behav*. 1983;24(4):385-396. 10.2307/2136404 6668417

[77] Bromberger JT, Matthews KA. A longitudinal study of the effects of pessimism, trait anxiety, and life stress on depressive symptoms in middle-aged women. *Psychol Aging*. 1996;11(2):207-213. 10.1037/0882-7974.11.2.207 8795049

[78] Hernández-Acosta J . Encuesta Mundial De Valores para Puerto Rico: 2018. Instituto de Estadísticas de Puerto Rico; 2019: 1-122. www.estadisticas.pr

[79] Williams DR, Yan Y, Jackson JS, et al. Racial differences in physical and mental health: socio-economic status, stress, and discrimination. *J Health Psychol*. 1997;2(3):335-351. 10.1177/135910539700200305 22013026

[80] Hughes ME, Waite LJ, Hawkley LC, et al. A short scale for measuring loneliness in large surveys: results from two population-based studies. *Res Aging*. 2004;26(6):655-672. 10.1177/0164027504268574 18504506 PMC2394670

[81] Messina CR, Lane DS, Glanz K, et al. Relationship of social support and social burden to repeated breast cancer screening in the Women's Health Initiative. *Health Psychol*. 2004;23(6):582-594. 10.1037/0278-6133.23.6.582 15546226

[82] Chen E, McLean KC, Miller GE. Shift-and-persist strategies: associations with socioeconomic status and the regulation of inflammation among adolescents and their parents. *Psychosom Med*. 2015;77(4):371-382. 10.1097/PSY.0000000000000157 26167560 PMC5890430

[83] Scheier MF, Carver CS, Bridges MW. Distinguishing optimism from neuroticism (and trait anxiety, self-mastery, and self-esteem): a reevaluation of the life orientation test. *J Pers Soc Psychol*. 1994;67(6):1063-1078. 10.1037/0022-3514.67.6.1063 7815302

[84] Alegría M, Jackson JS, Kessler RC, et al. Collaborative Psychiatric Epidemiology Surveys (CPES), 2001-2003 [United States]. Inter-university Consortium for Political and Social Research; 2024. 10.3886/ICPSR20240.v9

[85] Underwood LG, Teresi JA. The daily spiritual experience scale: development, theoretical description, reliability, exploratory factor analysis, and preliminary construct validity using health-related data. *Ann Behav Med*. 2002;24(1):22-33. 10.1207/S15324796ABM2401_04 12008791

[86] Loustalot FV, Wyatt SB, Boss B, et al. Psychometric examination of the daily spiritual experiences scale. *J Cult Divers*. 2006;13(3):162-167. 16989254

[87] Walen H, Lachman M. Social support and strain from partner, family, and friends: costs and benefits for men and women in adulthood. *J Soc Pers Relat*. 2000;17(1):5-30. 10.1177/0265407500171001

[88] Garcia DO, Wertheim BC, Manson JE, et al. Relationships between dog ownership and physical activity in postmenopausal women. *Prev Med*. 2015;70:33-38. 10.1016/j.ypmed.2014.10.030 25449694 PMC4274243

[89] Mujahid MS, Diez Roux AV, Morenoff JD, et al. Assessing the measurement properties of neighborhood scales: from psychometrics to ecometrics. *Am J Epidemiol*. 2007;165(8):858-867. 10.1093/aje/kwm040 17329713

[90] Hirsch JA, Moore KA, Barrientos-Gutierrez T, et al. Built environment change and change in BMI and waist circumference: Multi-Ethnic Study of Atherosclerosis. *Obesity*. 2014;22(11):2450-2457. 10.1002/oby.20873 25136965 PMC4224985

[91] Meyer J, Novak M, Hamel A, et al. Extraction and analysis of cortisol from human and monkey hair. *J Vis Exp*. 2014;83(83):e50882. 10.3791/50882-v PMC4089402 24513702

[92] Tucker KL, Bianchi LA, Maras J, et al. Adaptation of a food frequency questionnaire to assess diets of Puerto Rican and non-Hispanic adults. *Am J Epidemiol*. 1998;148(5):507-518. 10.1093/oxfordjournals.aje.a009676 9737563

[93] Palacios C, Trak MA, Betancourt J, et al. Validation and reproducibility of a semi-quantitative FFQ as a measure of dietary intake in adults from Puerto Rico. *Public Health Nutr*. 2015;18(14):2550-2558. 10.1017/S1368980014003218 25621587 PMC4955524

[94] Hispanic Community Health Study/Study of Latinos . HCHS/SOL Manual 15 Endpoint Ascertainment Procedures. Accessed February 3, 2023. https://sites.cscc.unc.edu/hchs/manuals-forms

[95] Rosal MC, White MJ, Borg A, et al. Translational research at community health centers: challenges and successes in recruiting and retaining low-income Latino patients with type 2 diabetes into a randomized clinical trial. *Diabetes Educ*. 2010;36(5):733-749. 10.1177/0145721710380146 20729512

[96] Wilcox S, Shumaker SA, Bowen DJ, et al. Promoting adherence and retention to clinical trials in special populations: a Women's Health Initiative workshop. *Control Clin Trials*. 2001;22(3):279-289. 10.1016/S0197-2456(00)00130-6 11384790

[97] National Institutes of Health . Final NIH Policy for Data Management and Sharing (NOT-OD-21-013). 2021. Accessed January 10, 2023. https://grants.nih.gov/grants/guide/notice-files/NOT-OD-21-013.html

[98] Greenland S, Pearl J, Robins JM. Causal diagrams for epidemiologic research. *Epidemiology*. 1999;10(1):37-48. 10.1097/00001648-199901000-00008 9888278

[99] Textor J, Hardt J, Knuppel S. DAGitty: a graphical tool for analyzing causal diagrams. *Epidemiology*. 2011;22(5):745. 10.1097/EDE.0b013e318225c2be 21811114

[100] Battaglia MP, Hoaglin DC, Frankel MR. Practical considerations in raking survey data. *Surv Pract*. 2009;2(5):1-10. 10.29115/SP-2009-0019

[101] Deville JC, Sarndal CE, Sautory O. Generalized raking procedures in survey sampling. *J Am Stat Assoc*. 1993;88(423):1013-1020. 10.1080/01621459.1993.10476369

[102] Kish L . Survey Sampling. John Wiley; 1965.

[103] US Census Bureau . Puerto Rico Community Survey. Accuracy of the data. https://www2.census.gov/programs-surveys/acs/tech_docs/accuracy/PRCS_Accuracy_of_Data_2021.pdf. Accessed August 19, 2023. 2021.

[104] Tucker KL, Mattei J, Noel SE, et al. The Boston Puerto Rican Health Study, a longitudinal cohort study on health disparities in Puerto Rican adults: challenges and opportunities. *BMC Public Health*. 2010;10(1):107. 10.1186/1471-2458-10-107 20193082 PMC2848197

[105] Mattei J, Tamez M, Rios-Bedoya CF, et al. Health conditions and lifestyle risk factors of adults living in Puerto Rico: a cross-sectional study. *BMC Public Health*. 2018;18(1):491. 10.1186/s12889-018-5359-z 29650018 PMC5898045

[106] Perez CM, Guzman M, Ortiz AP, et al. Prevalence of the metabolic syndrome in San Juan. *Puerto Rico Ethn Dis*. 2008;18(4):434-441. 19157247 PMC2717036

[107] Perez CM, Ortiz AP, Guzman M, et al. Distribution and correlates of the metabolic syndrome in adults living in the San Juan metropolitan area of Puerto Rico. *P R Health Sci J*. 2012;31(3):114-122. 23038883 PMC3469162

[108] Perez CM, Sanchez H, Ortiz AP. Prevalence of overweight and obesity and their cardiometabolic comorbidities in Hispanic adults living in Puerto Rico. *J Community Health*. 2013;38(6):1140-1146. 10.1007/s10900-013-9726-5 23846388 PMC3823746

[109] Perez CM, Soto-Salgado M, Suarez E, et al. High prevalence of diabetes and prediabetes and their coexistence with cardiovascular risk factors in a Hispanic community. *J Immigr Minor Health*. 2015;17(4):1002-1009. 10.1007/s10903-014-0025-8 24781780 PMC4214903

[110] National Heart Lung and Blood Institute . Charting the future together: the NHLBI Strategic Vision. Accessed January 10, 2023. https://www.nhlbi.nih.gov/about/strategic-vision

[111] US Census Bureau . Puerto Rico Community Survey 2021 one-year PUMS file. Accessed on February 18, 2023. https://www.census.gov/programs-surveys/acs/microdata/access.2021.html#listtab-735824205

